# Towards predicting individual cognitive trajectories in AD using Quantitative Systems Pharmacology

**DOI:** 10.1002/alz.087673

**Published:** 2025-01-09

**Authors:** Ivo Schuttel, Shaina M Short, Hugo Geerts

**Affiliations:** ^1^ Certara UK Limited, Sheffield, S1 United Kingdom; ^2^ Certara, Park City, UT USA; ^3^ Certara SimCyp, Berwyn, PA USA

## Abstract

**Background:**

While a number of recent anti‐amyloid antibodies demonstrated a robust reduction of amyloid biomarkers in clinical trials, the impact on functional improvement is much more variable. We hypothesize that this larger variability is driven by comedications, common genotype variants and underlying tau pathology.

**Method:**

In a previously calibrated computational neuroscience model of ADAS‐Cog, we implemented the effect of soluble amyloid monomers and oligomers on glutamate and nicotinic AChR neurotransmission and the effect of intracellular tau oligomers on voltage‐gated Na and K+ channels and synaptic density. The quantitative relationship between amyloid and tau biomarker was derived from a mechanistic QSP model. The effect of CNS active comedications and impact of COMTVal156Met and 5HTT‐rs23352 genotype on relevant neurotransmitter receptors were calculated using affinity data and clinical information.

**Result:**

We used a virtual twin approach to simulate the cognitive trajectory of individual patients from the ADNI database based on their individual baseline comedications, genotypes, amyloid and tau load. Comparison to the actual observed trajectory revealed the importance of common dopaminergic and serotonergic genotypes. We also found that serotonergic pharmacology of comedications could substantially modulate differential cognitive trajectories for the same amount of amyloid increase.

**Conclusion:**

Such a predictive platform, once validated can be useful to support clinical trial design, with regard to stratification and protocol amendments and reducing outcome variability by comparing the cognitive trajectory of subjects on active treatment with the same patients in a virtual placebo arm.

